# Is polio making a comeback? The cost of vaccine hesitancy and the disparity in vaccine coverage

**DOI:** 10.17179/excli2023-6594

**Published:** 2023-10-26

**Authors:** Thalles Guilarducci Costa, Rizia Rocha Silva, João Victor Rosa de Freitas, Rodrigo Luiz Vancini, Marilia Santos Andrade, Claudio Andre Barbosa de Lira

**Affiliations:** 1 Federal University of Goiás, Goiânia, Brazil; 2 Federal University of Espírito Santo, Vitória, Brazil; 3 Federal University of São Paulo, São Paulo, Brazil

## ⁯⁯

Poliomyelitis is a disabling and life-threatening disease caused by the poliovirus, which can result in paralytic paralysis. The virus spreads primarily from person to person, and it is highly contagious, affecting mostly children under five years with the potential to cause irreversible paralysis (in 5-10 % of the cases) (WHO, 2020[23]). In the early 1950s, the first polio vaccine (the inactivated poliovirus vaccine, i.e., IPV) was announced to the world. However, the oral polio vaccine (OPV), developed in 1962, is now the most used vaccine to prevent the disease (Thompson, 2022[19]), containing live-attenuated poliovirus. When compared with the parenteral inactivated poliovirus vaccine (IPV), it is feasible and low cost and provides higher immunization rates (Cooper et al., 2022[3]). Nonetheless, the genetically modified poliovirus present in the OPV can evolve during replication (mutation) and revert to wild-type characteristics, leading to outbreaks of circulating vaccine-derived poliovirus (cVDPVs) (Cooper et al., 2022[3]). In this case, in low immunization coverage areas, the poliovirus can circulate long enough to regain wild-type virulence with transmissibility (Tebbens et al., 2006[18]) and illness potential, leading to vaccine-associated paralytic poliomyelitis (VAPP) (Dowdle et al., 2003[6]). Because of this issue, most high-income countries have replaced OPV with IPV, but the latter's high cost makes it difficult for lower and middle-income countries to meet this requirement. As a result, the VAPP burden is primarily concentrated in low-income and middle-income countries (Platt et al., 2014[17]), particularly in Sub-Saharan Africa and East Asia (Modlin et al., 2021[13]). Unfortunately, polio has emerged in some developed countries where it was thought to be eradicated for decades (Thrush, 2022[20]). For example, the virus had not been detected in the United States since 1979, the American continent since 1994, and the United Kingdom since 2002 (McKenna 2022[12]), but it has now been discovered in wastewater surveillance in some regions of the United States and London sewage (Klapsa et al., 2022[9]; New York State, 2022[14]). More specifically, on February 8, 2022, in London during sewage treatment, more than 118 polioviruses were isolated (Klapsa et al., 2022[9]). Individuals who did not receive vaccination or whose vaccination was incomplete may have contracted the virus through local transmission rather than travel to endemic areas (New York State, 2022[14]). Therefore, despite the World Health Organization declaring the natural poliovirus form (type 2) to be globally eradicated in 2015, with only two remaining countries with endemic status (Pakistan and Afghanistan), the current low rates of global childhood vaccination pose a real problem for polio prevention (WHO, 2016[21]). Vaccination coverage has fallen below 80 % in almost all countries in the Americas in recent years, and four of them are at extremely high risk of wild poliovirus reintroduction: Brazil, Haiti, Peru, and the Dominican Republic (OPAS, 2022[15]). Not only cVDPVs but also wild types have been discovered in Pakistan, Afghanistan, Malawi, and Mozambique, and polio cases have been increasing since 2015 (Dattani et al., 2022[5]; WHO, 2022[24]). Until poliovirus transmission is stopped, all countries are at risk of polio importation, particularly vulnerable countries with poor public health and immunization services and travel or trade links to endemic countries (WHO, 2022[22]).

Vaccination has faced opposition despite the obvious fact that it saves lives and reduces human suffering (Albrecht, 2022[1]). Vaccine hesitancy is defined as a delay in accepting or refusing vaccination despite the availability of vaccination services (Dubé et al., 2013[7]). This reluctance has grown stronger as a result of the spread of fake news on the internet and social media (Albrecht, 2022[1]), and it is associated with low education, literacy, and economic status, doubts about the effectiveness of current vaccines, concerns about their short-term or long-term effects, doubts about the real usefulness of administering so many vaccines in early childhood (Dubé et al., 2013[7]).

The commemoration of 32 years of polio eradication in Brazil, in particular, has brought a bitter taste of vaccine coverage further away from the target of protected children, which was last achieved in 2015 (OPAS, 2022[15]). The current vaccination campaign only reached 67 % of the target population, and the booster dose only reached 52 % of the population (Dandara, 2022[4]). Vaccination hesitancy in the country has worsened in the face of the COVID-19 pandemic, owing to the widespread dissemination of fake news and disinformation caused by Brazilian political polarization, and has been exacerbated by opposing positions taken by President Jair Messias Bolsonaro and the Brazilian scientific community, which can be cited as crucial in discouraging adherence to vaccination campaigns, such as polio (Galhardi et al., 2022[8]).

Given that polio infections are asymptomatic (Cohen, 2004[2]; WHO, 2020[23]), a large number of people may be unknowingly transmitting the polio virus. The clinical features range from asymptomatic infection to paralysis in the most severe cases. There is some functional recovery after the acute phase of the disease, but in the long run, survivors of the paralytic form of polio can develop problems due to the disease, such as weakness, fatigue, and atrophy, and these symptoms can emerge 15-30 years after functional recovery, as known as post-polio syndrome, reaching 15 %-80 % of polio survivors (Koopman et al., 2015[10]; Lo and Robinson, 2018[11]). Therefore, health authorities must promote and redouble public awareness campaigns for the population on the importance of vaccinating children, particularly to complete the vaccination routine, which consists of four doses between the ages of 6 weeks and 6 years. Failure to complete the vaccination routine allows the virus to replicate and regain virulence. We cannot afford to relax our guard. 

Unfortunately, the vaccine hesitancy is not a problem only for poliomyelitis. The current health emergency caused by the COVID-19 pandemic shows that many people refused to be vaccinated (Wiysonge et al., 2022[25]). In 2021, regional coverage with the first dose of the vaccine that protects against measles, rubella, and mumps was 85 %. Only six countries achieved optimal coverage of 95 % or greater to sustain the elimination of these diseases, and ten countries reported less than 80 % coverage (PAHO, 2023[16]). This is a complex problem that emerges from fake news about potential problems that the vaccine could cause at long term. To face this situation, some actions are recommended:

- Promotion of awareness campaigns about the benefits of vaccines among people
- Strengthen World Health Organization's immunization agenda with the objective is to build strong immunization programs around the world, strengthen existing programs, and eliminate inequality
- Raise awareness among health authorities, especially in low- and middle-income countries, of the importance of vaccines
- Encourage social media managers (Instagram, Facebook, etc.) to fight the spread of fake news about vaccines.

Considering current data on polio vaccination coverage, the return of polio is approaching reality. Polio epidemics throughout history claimed lives and left sequelae. Therefore, it is the duty of all of society to prevent history from repeating itself.

## Declaration

### Author contributions

TGC led the conceptualization, research and writing of the article. RRS, JVRF, RLV, MSA and CABL contributed to manuscript writing and revisions. All authors approved the final version of the manuscript.

### Conflict of interest

The authors declare that the article has been written in the absence of any commercial or financial relationships that could imply a potential conflict of interest.
